# Supervised machine learning-based prediction of modern contraceptive use among sexually active women in Nepal

**DOI:** 10.1371/journal.pdig.0001578

**Published:** 2026-07-13

**Authors:** Jibesh Acharya, Divya Shakya

**Affiliations:** Population and Statistics Research Hub, Lalitpur, Nepal; Nanjing Medical University Affiliated Wuxi People’s Hospital: Wuxi People’s Hospital, CHINA

## Abstract

Modern contraceptives play a key role in reducing maternal and infant mortality, preventing unsafe abortions, and advancing women’s social and economic participation. Despite their importance, contraceptive use remains uneven across subpopulations in Nepal. This study aimed to identify key predictors of modern contraceptive use among sexually active women using supervised machine learning and data from the 2022 Nepal Demographic and Health Survey. A total weighted sample of 7,090 women residing with their partners was analyzed. Four supervised machine learning models, Random Forest, Extreme Gradient Boosting, Support Vector Machine, and Logistic Regression, were trained on a balanced dataset. Hyperparameters were optimized through Bayesian optimization. Model performance was assessed on an independent test set. The area under the receiver operating characteristic curve was model evaluation metric. Shapley Additive Explanations quantified predictor importance. The prevalence of modern contraceptive use was 53.70%. Random Forest model achieved the best predictive performance, with highest accuracy and an area under the curve. The most important predictors were fertility preference (0.0737), children ever born (0.0291), secondary or higher education (0.0214), internet use (0.0211), having at least one son (0.0203), contraceptive decision-making (0.0195), Janajati ethnicity (0.0181), age 35–49 years (0.0176), current employment (0.0169), and pregnancy loss history (0.0151). Women who did not desire another child, had children, used the internet, were employed, belonged to the Janajati ethnic group, aged 35–49 years, or had experienced pregnancy loss were more likely to use modern contraceptives. Women who desired another child, lacked formal education, or made contraceptive decisions independently showed a lower likelihood of use. Thus, modern contraceptive use is jointly shaped by demographic, reproductive, and socioeconomic factors. Interventions should prioritize younger women and adolescents, expand digital reproductive health communication, encourage couple engagement in contraceptive decision-making, and implement culturally tailored programs to address disparities across sub-populations.

## Author summary

A range of personal, social, and cultural factors influences decisions about contraceptive use. In Nepal, modern contraceptives use varies across different groups of women, and understanding these differences can help improve family planning programs. We used a machine learning approach to identify the factors associated with modern contraceptive use in Nepal from a Demographic and Health survey of 2022. A woman’s desire for more children was the strongest predictor of contraceptive use. Women who had children, used the internet, were employed, were aged 35–49 years, or had experienced pregnancy loss were more likely to use modern contraceptives, whereas women who desired another child, had no formal education, or made contraceptive decisions independently were less likely to use modern methods. Women from the Janajati ethnic group showed a slightly higher likelihood of use overall, although this varied across regions. These findings suggest that family planning programs in Nepal should prioritize reaching younger women earlier, engage male partners in reproductive health decisions, and expand the use of digital platforms to deliver information. Targeted efforts are also needed to support women with no formal education and to address cultural norms that influence contraceptive behavior in different communities.

## Background

Modern contraception has emerged as a cornerstone of public health advancement, profoundly shaping reproductive health outcomes over the past century. It has played a pivotal role in reducing maternal and infant mortality, preventing unsafe abortions, and expanding women’s opportunities to pursue education, employment, and social participation. These clinically approved measures—oral contraceptive pills, injectables, intrauterine devices (IUDs), implants, vaginal rings, condoms, and male and female sterilization—are significant in empowering individuals and couples to plan and space their pregnancies, preventing unintended pregnancies and promoting healthy birth intervals, lowering perinatal risks, and improving maternal and child well-being [1]. Beyond its health benefits, family planning has far-reaching social benefits. It supports gender equality, economic stability, and educational attainment. In Nepal, where reproductive health remains a national development priority, modern contraception is critical for promoting health equity and meeting long-term population and health goals [2].

Currently, the global modern contraceptive prevalence rate (mCPR) is approximately 49% among women of reproductive age (15–49 years). While usage exceeds 70% in parts of Europe and North America, it remains below 35% in sub-Saharan Africa and some South Asian contexts. Urban–rural disparities, educational attainment, income levels, and sociocultural norms continue to influence contraceptive uptake across and within countries. According to UNDESA, an estimated 874 million women use modern contraception, nearly double the number in 1990. Usage is highest among women aged 25–44 and lowest among adolescents and young adults [3].

**Fig 1:**
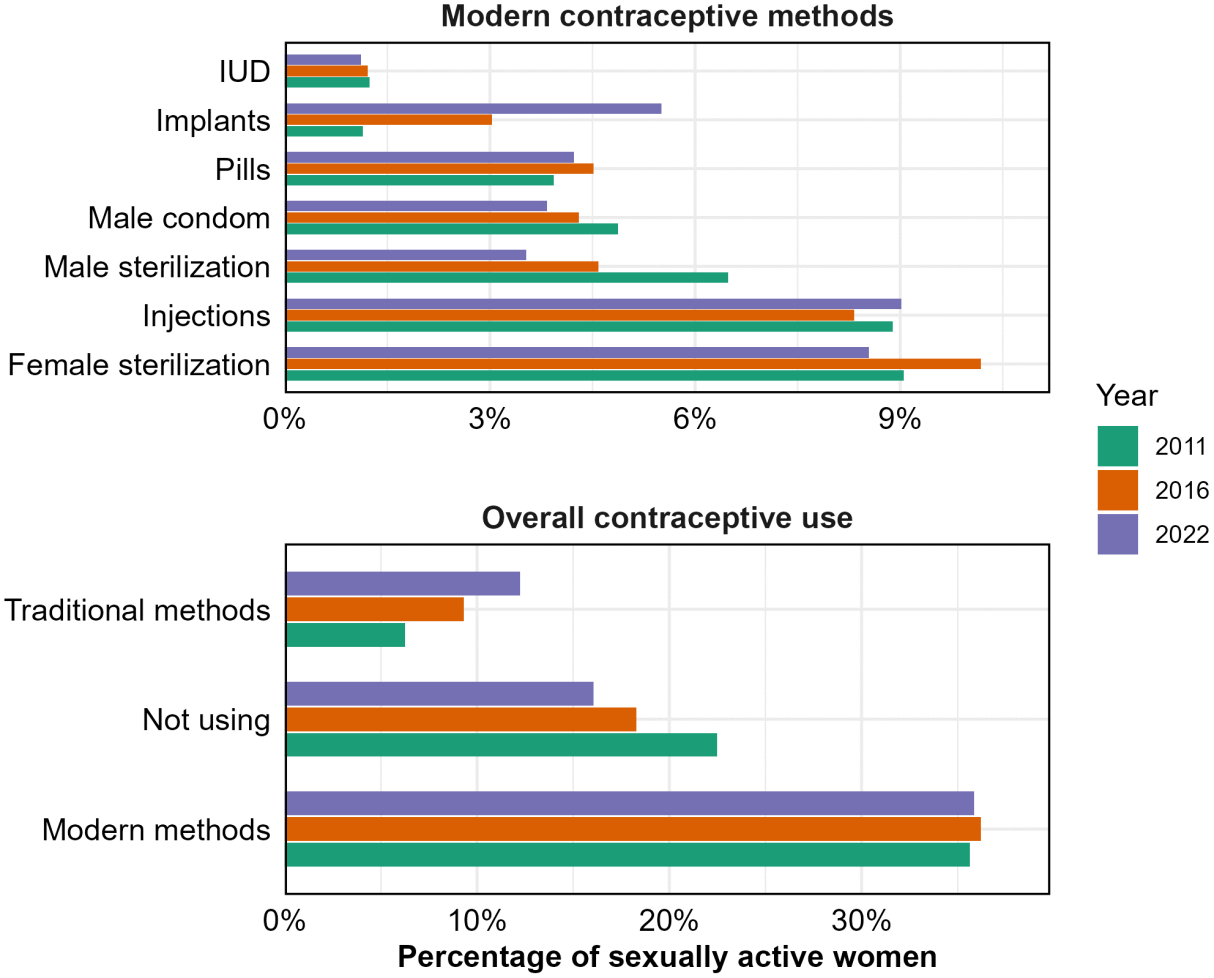
Percentage distribution of contraceptive use and methods among sexually active women.

In Nepal, modern contraceptive use has seen a modest improvement due to the national health policies and community-based interventions. The National Health Report 2022/23 reported an mCPR of 43%, with policy goals to raise this figure to 60% by 2030. Concurrently, the unmet need for contraception is targeted for reduction from 21% to 10% [4]. However, contraceptive use remains comparatively low among adolescents and young women compared to that of older women, a disparity attributed to limited autonomy, inadequate access to youth-friendly reproductive health services [5–7]. While the national mCPR has seen some progress, the overall use of modern contraceptives among sexually active women over the last decade has remained relatively stable at around 36% over the last decade (Fig 1). During this period, the use of traditional methods nearly doubled, from 6% to 12%, while the reliance on male-focused methods, such as condoms, and male sterilization declined. Among female-focused modern methods, uptake of implants increased markedly (from 1% to 6%), injectables remained stable at around 9%, and the use of oral pills rose slightly.

Nepal has implemented several policies to improve access to a comprehensive range of modern contraceptive methods, including long-acting reversible contraception, and reduce discontinuation [2]. Many studies have explored the association between contraceptive use and various sociodemographic and behavioral factors. Age [8], the educational attainment of both partners [9,10], women’s autonomy in reproductive decision-making [11] have all been identified as significant predictors. Household wealth [12], employment status and broader socioeconomic conditions also shape the use of contraceptives. Understanding these dynamics is vital for shaping effective family planning strategies.

To address this gap, the present study applies supervised machine learning models to data from the Nepal Demographic and Health Survey (NDHS) of 2022 to predict modern contraceptive use among sexually active women. The study also incorporates the SHapley Additive exPlanations (SHAP), to interpret the model’s predictions and identify the important factors associated with contraceptive behavior. By integrating predictive accuracy with interpretability, this research seeks to generate actionable insights to guide evidence-based policy making, improve targeting of reproductive health interventions,

## Methods

### Ethics statement

Data for this study were from the Nepal Demographic and Health Survey 2022, which was reviewed by the Nepal Health Research Council (NHRC) and the ICF Institutional Review Board. Written informed consent was obtained from all participants before their inclusion in the study.

### Study design

The study used a secondary dataset from the 2022 Nepal Demographic and Health Survey (NDHS), a nationally representative cross-sectional survey. The analysis was based on the women’s dataset (IR file). During the survey, data collection was conducted between January 5 and June 22, 2022, by New ERA in collaboration with the Ministry of Health and Population (MoHP).

A total of 14,845 women aged 15–49 (8019 from urban and 6826 from rural) were surveyed [13]. The NDHS included questions about recent sexual activity, and women reporting no sexual activity were excluded from the analysis. The final analytical sample consisted of 7090 weighted women who were sexually active within the past four weeks and were in a union during the study period. The dataset was accessed with authorization from the DHS Program (http://www.dhsprogram.com). Additional details are available in the final NDHS 2022 report, accessible at The DHS Program - Nepal: DHS, 2022 - Final Report (English).

### Study variables

#### Dependent variable.

The outcome variable was modern contraceptive use (MCU), defined as a dichotomous variable. Women currently using a modern contraceptive measure were coded 1 (yes), and those not using any modern method were coded as 0 (no).

#### Independent variables.

A total of 21 independent variables were selected from the dataset, informed by relevant literature and prior findings [14–18]. These included: current age, age at marriage, education, occupation, ethnicity, religion, age at first sex, Children Ever Born (CEB), number of living daughters and sons, exposure to family planning advertisements, decision maker for using contraception, fertility preference, pregnancy losses, husband’s education, internet usage, own a mobile phone, time to reach the nearest health facility, wealth quintile, place of residence, and province. (see S1 Table).

### Data processing and machine learning analysis

This study employed a supervised Machine Learning (ML) approach, in which models learn patterns from training data to generate predictions for new observations [19]. A structured framework from Yufeng Guo’s seven-step methodology for ML: data collection, data preparation, model selection, model training, model evaluation, hyperparameter tuning, and prediction, was adopted [14,20].

All analyses were implemented in Python (version 3.11) within the JupyterLab environment, utilizing the scikit-learn (version 1.2.2) imbalanced-learn, scikit-optimize, and SHAP libraries.

### Data preparation and preprocessing

Data preprocessing is a foundational step that influences model performance and includes data cleaning, feature engineering, and dataset partitioning [16]. Outliers were identified and addressed, and variables with sparse categories, such as current age, age at marriage, education, ethnicity, religion, age at first sex, and occupation, were recoded or grouped accordingly. Composite variables, including exposure to family planning advertisements, number of living sons, and number of living daughters, were derived from existing variables (S1 Table).

### Feature engineering

Categorical variables were transformed into numerical representations suitable for ML algorithms. One-hot encoding was applied to nominal variables using *Scikit-learn’s ColumnTransformer* in combination with *OneHotEncoder*, generating binary indicator columns for each category (1 = present, 0 = absent). The first category was dropped to avoid multicollinearity.

All preprocessing steps were fitted on the training dataset and subsequently applied to the test dataset to prevent data leakage.

### Data splitting and class imbalance handling

The dataset was split into training (80%) and testing (20%) sets using stratified random sampling, ensuring the class distribution of the outcome variable was preserved across both sets [21].

To address class imbalance, the Synthetic Minority Oversampling Technique (SMOTE) was applied to the training data. SMOTE generates synthetic minority-class observations by interpolating between existing samples in the feature space, reducing bias toward the majority class without duplicating observations.

### Multicollinearity assessment

Multicollinearity among predictors was assessed using the Variance Inflation Factor (VIF) on the training dataset. The mean VIF across predictors was approximately 4, indicating low overall multicollinearity. Although some dummy variables showed high VIF values (CEB-1 or more, religion-Hindu, and sons-Yes), this is expected in one-hot encoded data and does not substantially affect tree-based models (see S2 Table). Therefore, all variables were retained.

### Feature selection

Feature selection was performed using the Boruta algorithm, a Random Forest–based wrapper method that compares the importance of each variable with randomized shadow features [22]. The algorithm was implemented using a Random Forest base estimator with 200 trees and a maximum depth of 7.

From 44 encoded features derived from the 21 variables, Boruta confirmed 21 features and identified 2 as tentative after 100 iterations. Mapping these back to the original variables, 19 of the 21 predictors were retained. Two variables (place of residence and religion) were excluded. For the final model training, the 23 Boruta-confirmed and tentative encoded features were used, reducing dimensionality by approximately 48%.

### Model selection

Four supervised ML algorithms were selected for comparative analysis: Logistic Regression (LR), Random Forest (RF), Extreme Gradient Boosting (XGB), and Support Vector Machine (SVM). These models are widely used in reproductive and public health research [23].

Logistic Regression models the probability of a binary outcome using the logistic function and is a highly interpretable baseline model [24]. Random Forest is an ensemble method that constructs multiple decision trees and aggregates their predictions, offering robustness to noise, reduced variance, and the ability to handle high-dimensional data [25,26]. XGBoost is a gradient boosting algorithm that minimizes a specified loss function (log loss for classification) using a gradient descent framework. It is efficient, handles missing data well, and includes regularization to reduce over-fitting [27]. SVM identifies an optimal hyperplane that maximizes the margin between classes and can model non-linear relationships using kernel functions [28,29].

### Model training and evaluation

All models were implemented within a pipeline framework, with SMOTE applied within cross-validation to prevent data leakage. Hyperparameter tuning was conducted using stratified 10-fold cross-validation on the training dataset. Model performance was evaluated on an independent test set using multiple metrics: area under the receiver operating characteristic curve (AUC), accuracy, sensitivity (recall), specificity, precision, and F1-score. AUC was the primary evaluation metric, as it assesses discrimination across all classification thresholds and is robust to class imbalance [30].

### Hyperparameter tuning

Hyperparameter optimization was performed using Bayesian optimization via the *BayesSearchCV* function from the *scikit-optimize* library, with stratified 10-fold cross-validation and ROC-AUC as the evaluation metric. This approach iteratively builds a probabilistic surrogate model of the objective function, balancing exploration of the hyperparameter space [31].

For Logistic Regression, the regularization strength (C), penalty type (L1 or L2), and class weights were tuned. For Random Forest, the number of trees (200–800), maximum depth (5–30), minimum samples to split (2–20), minimum samples per leaf (1–10), and the number of features considered at each split (sqrt or log2) were optimized. For XGBoost, the hyperparameters included the number of estimators, maximum depth, learning rate, subsample ratio, column subsampling ratio, and gamma. For SVM, the regularization parameter (C), kernel coefficient (gamma), and class weights were tuned. All models were optimized using 20 iterations of Bayesian search with a fixed random seed (123).

### Model interpretability

To enhance interpretability, SHAP (SHapley Additive exPlanations) values were computed for the best-performing model. SHAP is a game-theoretic approach that quantifies each feature’s contribution to the model predictions based on Shapley values. A *TreeExplainer* was used to efficiently compute exact SHAP values for the tree-based model.

Three complementary visualizations were generated: (i) a mean absolute SHAP bar plot to rank the top predictors by overall importance, (ii) a beeswarm plot to illustrate the distribution and direction of feature effects across observations, and (iii) a waterfall plot to explain individual predictions by decomposing them into additive feature contributions from the baseline to the final predicted probability [32].

**Fig 2:**
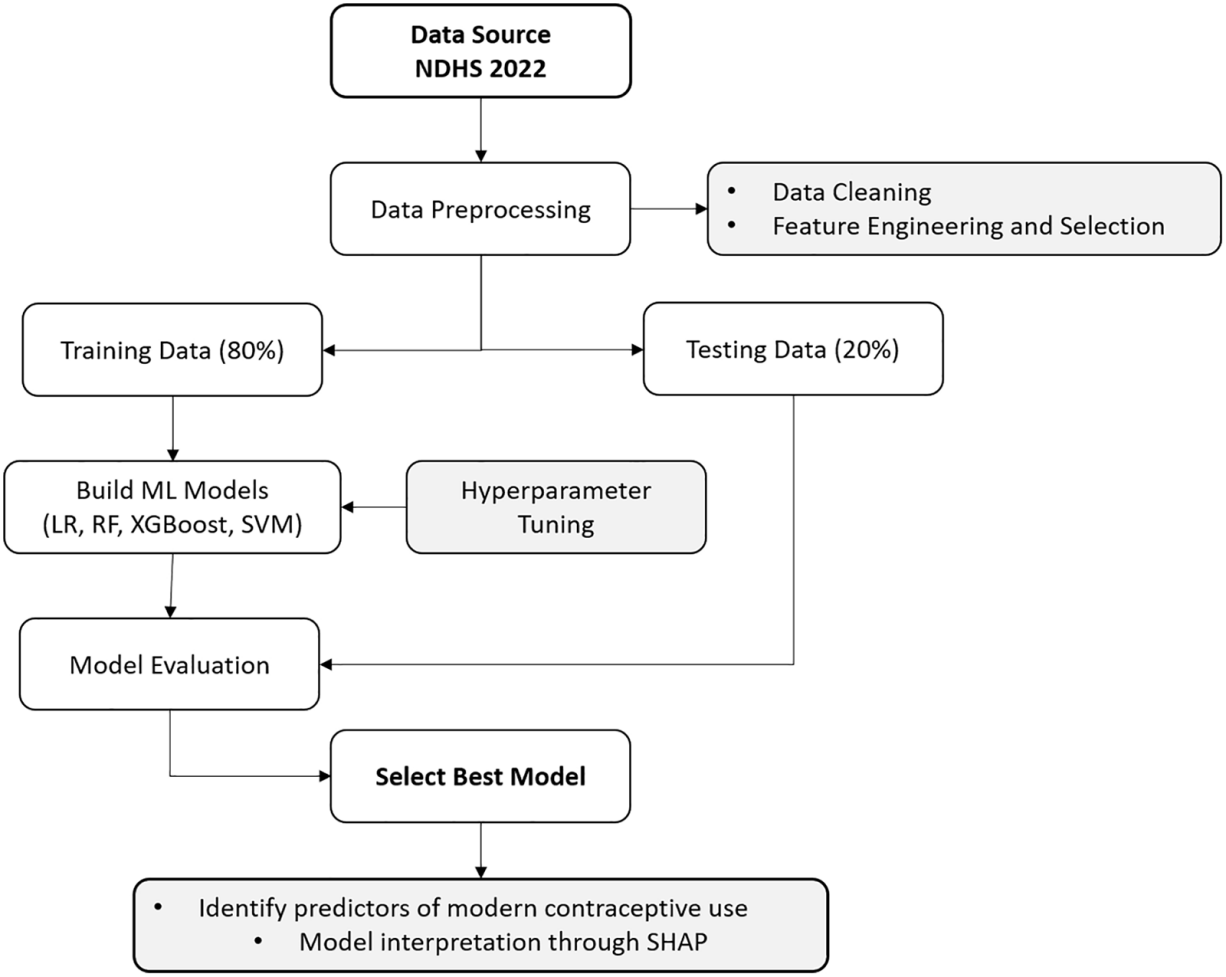
Study workflow diagram.

The overall study flow, including data preparation and analysis process, is illustrated in Fig 2.

## Results

### Socio-demographic characteristics and modern contraceptive use by respondents

The study included a total of 7090 (weighted) sexually active women of reproductive age. Overall, 3,807 women reported using modern contraceptive methods, corresponding to a prevalence of 53.70%, while 3,283 women (46.30%) were not using any modern methods.

The prevalence of modern contraceptives was high among women aged 35–49 years (n = 1926; 61.89%), those married before age 15 (n = 469; 65.23%), women with no education (n = 1394; 63.54%), employed women (n = 3106; 57.19%), and Janajati women (n = 1392; 58.61%). Similarly, women having their first sexual relationship before turning 15 years (n = 361; 63.78%), having at least one child ever born (n = 3732; 58.11%), with daughters (n = 1024; 62.25%) and sons (n = 3425; 58.06%), having access to family planning advertisements (n = 2583; 54.16%), taking self-decision for contraception (n = 626; 68.19%), with no fertility preference (n = 3327; 63.46%), without history of pregnancy losses (n = 2863; 54.19%), with husbands having basic education (n = 1627; 58.91%), never using internet (n = 1816; 62.47%), without a mobile phone (n = 836; 57.26%), living over 30 minutes from health facilities (n = 459; 60.08%), in the poorer wealth quintile (n = 815;59.17%), residing in rural areas (n = 1356; 59.32%), and from Sudurpaschim province (n = 339; 61.64%) had a higher prevalence of modern contraception (Table 1).

**Table 1:** Socio-demographic characteristics and modern contraceptive use by respondents.

Variables	Categories	Use of modern contraceptives	
		Yes (%)	No (%)
Current age (years)	15-24	428 (31.85)	916 (68.15)
	25-34	1453 (55.16)	1181 (44.84)
	35-49	1926 (61.89)	1186 (38.11)
Age at marriage	<15	469 (65.23)	250 (34.77)
	15-19	2385 (55.32)	1926 (44.68)
	20-24	784 (47.95)	851 (52.05)
	24+	170 (39.81)	257 (60.19)
Education	Basic	1271 (55)	1040 (45)
	No education	1394 (63.54)	800 (36.46)
	Secondary or above	1142 (44.2)	1442 (55.8)
Occupation	Not working	701 (42.28)	957 (57.72)
	Working	3106 (57.19)	2325 (42.81)
Ethnicity	Brahmin/Chettri	970 (51.16)	926 (48.84)
	Dalit	554 (55.79)	439 (44.21)
	Janajati	1392 (58.61)	983 (41.39)
	Madhesi	633 (51.13)	605 (48.87)
	Others	257 (43.78)	330 (56.22)
Religion	Buddhist	248 (55.11)	202 (44.89)
	Hindu	3209 (53.91)	2743 (46.09)
	Muslim	128 (41.97)	177 (58.03)
	Others	222 (58.12)	160 (41.88)
Age at first sex	<15	361 (63.78)	205 (36.22)
	15-19	2487 (55.59)	1987 (44.41)
	20-24	799 (48.39)	852 (51.61)
	24+	161 (40.35)	238 (59.65)
Children Ever Born	0	76 (11.38)	592 (88.62)
	1 or more	3732 (58.11)	2690 (41.89)
Number of living daughters	No	2784 (51.13)	2661 (48.87)
	Yes	1024 (62.25)	621 (37.75)
Number of living sons	No	382 (32.07)	809 (67.93)
	Yes	3425 (58.06)	2474 (41.94)
Exposure to family planning advertisements	Have Access	2583 (54.16)	2186 (45.84)
	No Access	1224 (52.74)	1097 (47.26)
Decision maker for using contraception	Husband	275 (44.43)	344 (55.57)
	Joint	2897 (52.57)	2614 (47.43)
	Others	9 (21.43)	33 (78.57)
	Self	626 (68.19)	292 (31.81)
Fertility preference	Don’t want	3327 (63.46)	1916 (36.54)
	Have another	397 (24.33)	1235 (75.67)
	Undecided	83 (38.79)	131 (61.21)
Pregnancy losses	No	2863 (54.19)	2420 (45.81)
	Yes	945 (52.3)	862 (47.7)
Husband’s education	Basic	1627 (58.91)	1135 (41.09)
	No education	598 (58.74)	420 (41.26)
	Secondary or above	1582 (47.79)	1728 (52.21)
Used internet	Never used	1816 (62.47)	1091 (37.53)
	Used	1992 (47.61)	2192 (52.39)
Own a mobile phone	No	836 (57.26)	624 (42.74)
	Yes	2971 (52.78)	2658 (47.22)
Time to reach the nearest health facility	30 min or less	3349 (52.93)	2978 (47.07)
	More than 30 min	459 (60.08)	305 (39.92)
Wealth quintile	Middle	813 (56.77)	619 (43.23)
	Poorer	815 (59.71)	550 (40.29)
	Poorest	680 (58.27)	487 (41.73)
	Richer	762 (48.66)	804 (51.34)
	Richest	738 (47.31)	822 (52.69)
Province	Bagmati	795 (51.89)	737 (48.11)
	Gandaki	306 (50.58)	299 (49.42)
	Karnali	248 (59.05)	172 (40.95)
	Koshi	659 (55.47)	529 (44.53)
	Lumbini	730 (55.64)	582 (44.36)
	Madhesh	730 (49.26)	752 (50.74)
	Sudurpaschim	339 (61.64)	211 (38.36)
Place of residence	Rural	1356 (59.32)	930 (40.68)
	Urban	2451 (51.02)	2353 (48.98)

### Balancing data and hyperparameter tuning

A mild class imbalance was observed in the outcome variable, with a higher proportion of women (53.70%) using modern contraceptives compared to non-users. To address this, the SMOTE was applied to the training dataset prior to model training. SMOTE generates synthetic observations for the minority class (women not using modern contraceptives), resulting in a more balanced class distribution. This approach enables the models to learn from a more equitable representation of both classes, thereby improving the stability and reliability of predictions.

Hyperparameters for all models were subsequently optimized to identify the best-performing configurations (see S3 Table).

### Predictive performance of machine learning models

The predictive performance of four machine learning models, Logistic Regression (LR), Random Forest (RF), Support Vector Machine (SVM), and Extreme Gradient Boosting (XGBoost), was evaluated on an independent test dataset. The primary evaluation metric was the area under the receiver operating characteristic curve (AUC), complemented by accuracy, sensitivity, specificity, precision, and F1-score.

**Fig 3:**
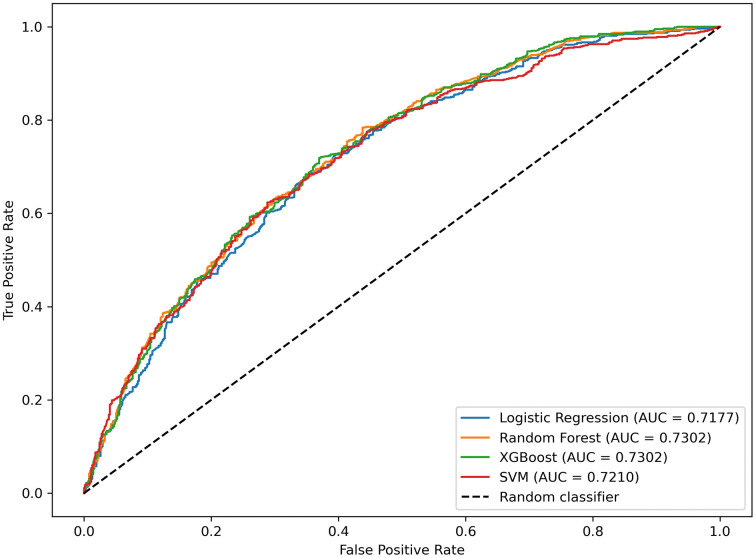
ROC curve of ML models.

Overall, all four ML models demonstrated comparable predictive performance, with AUC values ranging from 71.77% to 73.02% (Table 2, Fig 3). Random Forest and XGBoost achieved identical AUC values (73.02%), followed by SVM (72.10%) and Logistic Regression (71.77%).

**Table 2:** Performance metrics of machine learning models (Boruta-selected features).

Model	AUC (%)	Accuracy (%)	Sensitivity (%)	Specificity (%)	Precision (%)	F1-Score (%)
Random Forest	73.02	68.53	78.41	56.03	69.32	73.58
XGBoost	73.02	67.53	79.69	52.12	67.83	73.29
SVM	72.10	66.59	86.63	41.21	65.12	74.35
Logistic Regression	71.77	67.31	77.38	54.56	68.33	72.57

Random Forest showed better overall balance across evaluation metrics, with higher specificity (56.03% vs. 52.12%), precision (69.32% vs. 67.83%), and F1-score (73.58% vs. 73.29%) compared to XGBoost, indicating better identification of non-users of modern contraception (see S4 Table). In contrast, XGBoost achieved a slightly higher sensitivity (79.69% vs. 78.41%), reflecting a marginally better ability to identify contraceptive users.

Given its more balanced performance across multiple metrics, the Random Forest model was selected for subsequent interpretation and analysis, although differences between models were relatively small.

### Feature importance

**Fig 4:**
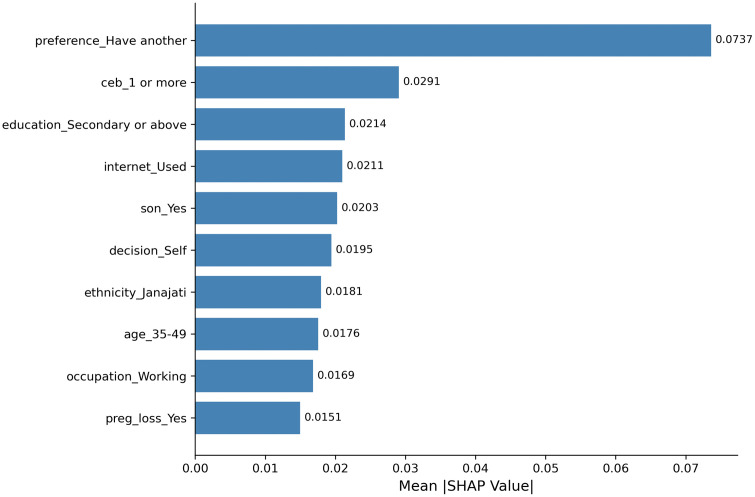
SHAP global importance plot of the optimized Random Forest model.

Global SHAP feature importance was used to identify the key predictors of modern contraceptive use from the optimized Random Forest model. This mean absolute SHAP value for each predictor quantifies its average contribution to the model’s output across all observations, regardless of direction. The ten most important predictors, ranked by mean absolute SHAP value, are presented in Fig 4.

Fertility preference for having another child was the most important predictor (mean |SHAP| = 0.0737), with an importance more than twice that of the second-ranked predictor. Having one or more children ever born ranked second (mean |SHAP| = 0.0291), followed by secondary or higher education (mean |SHAP| = 0.0214), internet use (mean |SHAP| = 0.0211), having at least one son (mean |SHAP| = 0.0203), self-decision-making for contraception (mean |SHAP| = 0.0195), Janajati ethnicity (mean |SHAP| = 0.0181), age 35–49 years (mean |SHAP| = 0.0176), current employment (mean |SHAP| = 0.0169), and history of pregnancy losses (mean |SHAP| = 0.0151) (see S5 Table).

### Model interpretation

**Fig 5:**
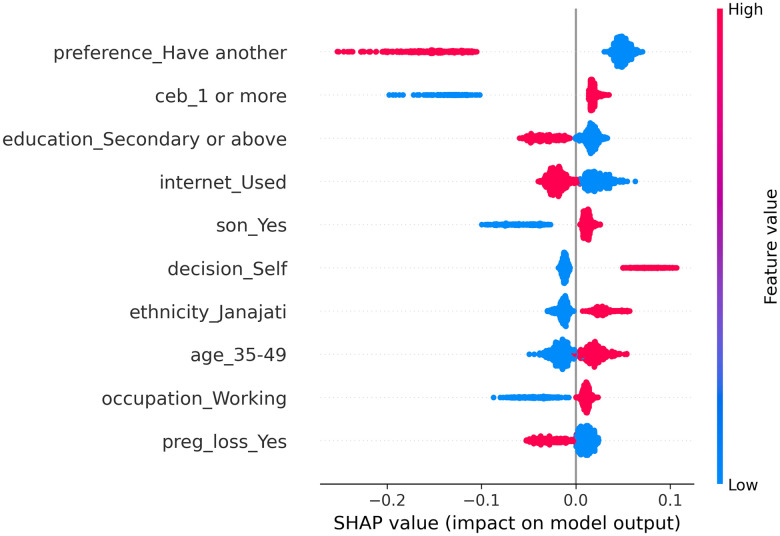
Beeswarm plot, ranked by mean absolute SHAP value generated by tuned RF model.

The beeswarm plot (Fig 5) illustrates both the direction and magnitude of each feature’s contribution to the predicted probability of modern contraceptive use across all observations. Each point represents an individual woman. Points to the right of the zero-reference line (positive SHAP value) indicate an increased predicted probability of modern contraceptive use, whereas points to the left indicate a decreased probability. Red hues represent high feature values, and blue hues represent low feature values for each predictor.

Fertility preference for having another child showed the strongest and most polarised pattern: women who desired another child (high value; red dots) had large negative SHAP values, while those who did not desire another child (low value; blue dots) clustered to the positive side, indicating a higher predicted probability of use. Having one or more children ever born showed a similar directional pattern, with the absence of children (blue dots) associated with strong negative contributions, extending to approximately −0.20.

Internet use, secondary or higher education, Janajati ethnicity, age 35–49 years, current employment, and history of pregnancy loss were generally associated with positive SHAP values for their higher levels, indicating increased predicted probability of modern contraceptive use. The absence of a son was associated with negative SHAP values, while the presence of a son showed modest and less consistent positive contributions near zero. Self-directed contraceptive decision-making showed a heterogeneous pattern, with a small subset of women exhibiting strongly positive SHAP values (approaching +0.11) and the remainder distributed near zero, yielding a mean SHAP value close to zero (−0.0010).

### Individual prediction

**Fig 6:**
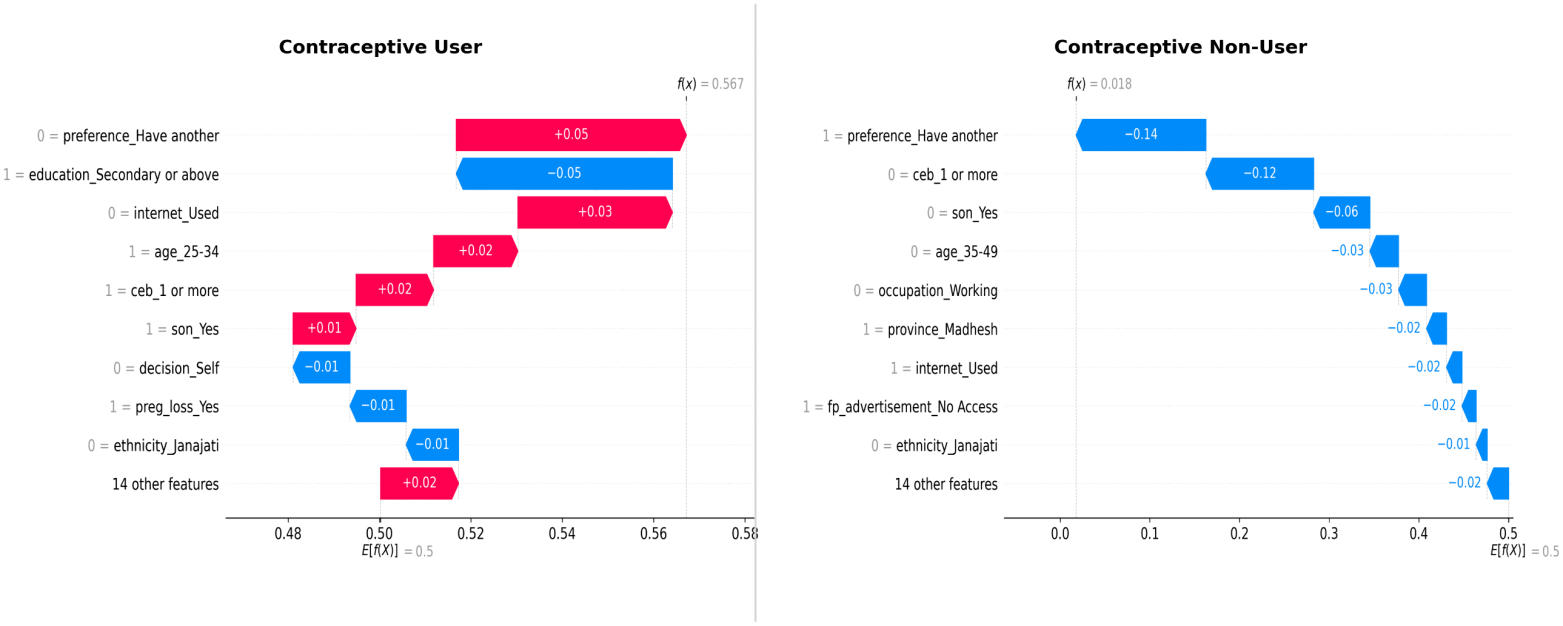
SHAP waterfall plot for individual predictions.

Figure 6 presents SHAP waterfall plots for two randomly selected, correctly classified women: one modern contraceptive user and another non-user. It illustrates how individual feature contributions shift the model’s baseline prediction (E[f(X)] = 0.5) to the final predicted probability.

For the contraceptive user, the baseline prediction increased to a final predicted probability of 0.567. The largest positive contributions were from not desiring another child (+0.05), not using the internet (+0.03), being aged 25–34 years (+0.02), having one or more children ever born (+0.02), and having a son (+0.01). In contrast, negative contributions were observed from having secondary or higher education (−0.05), not making self-directed contraceptive decisions (−0.01), a history of pregnancy loss (−0.01), and not belonging to the Janajati ethnic group (−0.01).

For the contraceptive non-user, the prediction decreased sharply to 0.018, indicating a very low likelihood of use. All features contributed negatively. The strongest effects were from desiring another child (−0.14), having no children yet (−0.12), absence of a son (−0.06), not being in the older reproductive age group (−0.03), not being employed (−0.03), residing in Madhesh Province (−0.02), using the internet (−0.02), having no access to family planning advertisements (−0.02), and not belonging to the Janajati ethnic group (−0.01).

Comparing the two cases, fertility preference, parity, and son preference were dominant drivers in both predictions, consistent with the global SHAP results. Notably, internet use contributed positively in one case but negatively in the other, highlighting that its effect is context-dependent and interacts with reproductive intentions and socio-demographic characteristics. This heterogeneity underscores that while population-level patterns are informative, individual predictions reflect a unique combination of characteristics that may not be fully captured by aggregate analyses.

## Discussion

This study applied machine learning models to identify key predictors of modern contraceptive use among women in Nepal, using nationally representative data from the 2022 Nepal Demographic and Health Survey. Among the four models evaluated, Random Forest demonstrated the best predictive performance (AUC: 73.02%; accuracy: 68.53%). This performance is comparable to similar machine learning studies in low- and middle-income countries, where AUC values typically range between 70% to 80% using Demographic and Health Survey data [14,33,34]. The moderate predictive performance observed in this study likely reflects the complexity of contraceptive behavior in Nepal, which is influenced by deeply embedded sociocultural factors—such as son preference, ethnic norms, and intra-household decision-making—that are not fully captured by survey-based variables.

SHAP analysis highlighted fertility preference, parity, education, internet use, son preference, contraceptive decision-making, Janajati ethnicity, age, employment, and a history of pregnancy loss as the key predictors, reflecting the multidimensional nature of contraceptive behavior.

Fertility intentions and family composition emerged as the most influential predictors. Women who did not want another child were more likely to use modern contraceptives, consistent with previous evidence in Nepal [1,2] and other countries [3–5]. Similarly, women with one or more children were more likely to adopt modern contraception, indicating that existing parity plays a crucial role in decisions related to birth spacing and limiting. The role of son preference was more nuanced: while having at least one son showed only a marginal association overall, its heterogeneous effects suggest that achieving a desired sex composition may reduce contraceptive demand for some women. This aligns with existing literature on son preference in Nepal [6–8].

Internet use was positively associated with modern contraceptive use, likely reflecting increased exposure to reproductive health information and awareness of contraceptive options. This finding is consistent with growing evidence on the role of digital platforms in shaping reproductive health behaviors in low- and middle-income settings [35,36].

Contraceptive decision-making dynamics further underscored the importance of partner involvement. Women who made decisions independently were slightly less likely to use modern contraceptives compared to those engaging in joint decision-making, supporting prior findings from Nepal [15]. This suggests that male partner involvement may facilitate contraceptive uptake, highlighting the importance of couple-centered approaches in family planning programs.

Ethnic variation was also evident. Women from the Janajati group showed a modestly higher likelihood of contraceptive use, consistent with some studies [15,37], although contrasting evidence exists [38]. These variations likely reflect contextual differences in cultural norms, geographic location, and socioeconomic conditions across populations [11].

Current employment was positively associated with modern contraceptive use, consistent with findings from prior studies [12,13]. Employed women may have greater economic independence and increased exposure to sexual and reproductive health information, which could motivate birth spacing or limiting to balance work and family responsibilities.

Women with a history of pregnancy loss were more likely to adopt modern contraceptives, consistent with a longitudinal cohort study in Nepal [14], suggesting increased contraceptive adoption following adverse reproductive outcomes.

Women aged 35–49 years were more likely to use contraceptives than younger women, while those aged 25–34 years showed a negative association. Lower contraceptive use among adolescents and younger women has been documented in prior studies [1,16]. The preference for sterilization after achieving the desired family size may further explain higher contraceptive use among older women.

Women with secondary or higher education showed a marginal positive association with modern contraceptive use, while those with no formal education showed a negative association, consistent with findings from multiple studies in Nepal [10]. This suggests that education may facilitate contraceptive awareness and adoption, though the relationship between higher education and contraceptive use may also be influenced by other factors such as delayed marriage or differing fertility intentions [20].

Collectively, these findings highlight the multidimensional nature of modern contraceptive use in Nepal, shaped by a complex interplay of fertility preferences, family composition, digital access, employment, ethnicity, age, and decision-making dynamics.

### Limitations and strengths of the study

This study has several limitations. First, Random Forest models are inherently less interpretable than conventional statistical models; however, the incorporation of SHAP analysis helped quantify predictor contributions and improve transparency. Second, the cross-sectional nature of the 2022 NDHS precludes causal inference between predictors and modern contraceptive use. Third, the model was developed on a single survey dataset without external validation, which may limit generalizability. Fourth, reliance on self-reported data introduces the possibility of recall and social desirability bias, particularly for sensitive reproductive health information. Fifth, although SMOTE addressed class imbalance, it generates synthetic observations that may introduce artificial variability into the training data. Finally, unmeasured confounders — including psychosocial factors, relationship dynamics, and community-level influences — may have affected the findings.

Despite these limitations, the study has several strengths. It applied a rigorous machine learning framework, including data balancing using SMOTE and Bayesian hyperparameter optimization, to improve model performance and reduce bias due to class imbalance. The use of an independent testing dataset strengthened the internal validity of the findings. Moreover, leveraging a nationally representative dataset enhances the relevance of the results for informing family planning programs and policy in Nepal.

## Conclusions

This study identified key predictors of modern contraceptive use among women in Nepal using a supervised machine learning approach. Random Forest demonstrated the best predictive performance and was used for SHAP-based interpretation.

The findings highlight fertility preference, parity, digital access, education, son preference, decision-making dynamics, ethnicity, age, employment, and reproductive history as key predictors. Women who did not desire additional children, had one or more children, were employed, had internet access, or had experienced pregnancy loss were more likely to use modern contraceptives. In contrast, women who desired additional children, lacked formal education, or made contraceptive decisions independently were less likely to use them. Older women were more likely to use contraception, while younger women showed lower uptake. The influence of ethnicity and son preference varied across contexts, reflecting underlying sociocultural dynamics.

These findings emphasize the need for targeted, context-sensitive family planning strategies in Nepal. Programs should prioritize reaching younger women and adolescents, expand digital health communication, and promote couple-based decision-making. Efforts to improve access among women with no formal education and to address culturally embedded norms are also critical.

Future research should focus on external validation of these models in other settings and incorporate longitudinal designs to better understand how fertility intentions, gender dynamics, and sociocultural factors evolve over time and influence contraceptive behavior.

## Supporting information

S1 Table Variable definitions. This file provides the operational definition of all variables included in this study, along with their summarization and categorization information.
(DOCX)

S2 Table Correlation matrix. This CSV file contains the correlation matrix of all predictor variables used in the study.
(CSV)

S3 Table Optimized hyperparameters of the Random Forest model. This CSV file contains the list of parameters optimized via hyperparameter tuning and their corresponding optimal values.
(CSV)

S4 Table Confusion matrix. This document presents the confusion matrix of the Random Forest model.
(DOCX)

S5 Table SHAP values of the Random Forest model. This CSV file contains the mean and absolute SHAP values of features obtained from the Random Forest model.
(CSV)
