# Supplementary material for: Supervised machine learning-based prediction of modern contraceptive use among sexually active women in Nepal
Source: PLOS Digit Health. 2026 Jul 13;5(7):e0001578. doi: 10.1371/journal.pdig.0001578 (PMC13362090; doi:10.1371/journal.pdig.0001578)
Supplement: S1 Table — This file provides the operational definition of all variables included in this study, along with their summarization and categorization information. (DOCX) [file pdig.0001578.s001.docx]

**Operational Definitions of variables in the study**

| **S.N.** | **Variables** | **Descriptions** |
| --- | --- | --- |
| 1 | Modern Contraceptive Use (MCU) | A dichotomous variable derived from the types of current contraceptives used by the respondent, categorised into Yes and No. |
| 2 | Current age (in years) | Self-reported age of respondents at the survey period; categorised into 15-24 years, 25-34 years, and 35-49 years |
| 3 | Age at marriage | Self-reported age at first marriage of respondents; categorised into less than 15 years, 15-19 years, 20-24 years, and 24 years and above. |
| 4 | Education | The highest level of education attained by respondents, summarized into No education (those who have never attended school and those in informal preschool), Basic education (those who have attended 0–4 grades or in school-based pre-primary class or have completed grade 8 at the lower basic level), and Secondary or above (completed grade 12 or above). |
| 5 | Occupation | Employment status of respondents, summarized into Working and Not Working. |
| 6 | Ethnicity | Ethnic groups of respondents: summarized to Brahmin/Chhetri (Hill Brahmin and Hill Chhetri), Dalit (Hill Dalit and Terai Dalit), Madhesi (Terai Brahmin/Chhetri and Other Terai caste), Janajati (Hill Janajati and Terai Janajati), and Others (Muslim, Newar, and others) |
| 7 | Religion | Religion of respondents: Hindu, Buddhist, Muslim, and Others |
| 8 | Age at first sex | Self-reported age at first sex of respondents; categorised into less than 15 years, 15-19 years, 20-24 years, and 24 years and above. |
| 9 | Children Ever Born (CEB) | Total number of children born to a respondent; categorised into 0 and 1 or more. |
| 10 | Number of living sons | A composite variable derived from the number of sons living in the same family and the number of sons living elsewhere, categorised into 0, and 1 or more. |
| 11 | Number of living daughters | A composite variable derived from the number of daughters living in the same family and the number of daughters living elsewhere, categorised into 0, and 1 or more. |
| 12 | Exposure to family planning advertisements | Summarised into two categories: No Access, and Have Access. Respondents who have heard or seen family planning advertisements on radio/TV/newspaper/magazine/text message on mobile phone/Facebook/Twitter/Instagram/Poster/Brochure/Outdoor Sign/Billboard/Community Meetings Events were considered to have access to such advertisements. |
| 13 | Decision maker for using contraception | Decision maker for using contraception among the couple, categorised into Self, Husband, Joint, and Others. |
| 14 | Fertility preference | Fertility preference of the respondent; categorised into Don’t want, Have another, and Undecided |
| 15 | Pregnancy losses | History of pregnancy losses of the respondent; categorised into Yes and No. |
| 16 | Husband’s education | The highest level of education attained by the respondent’s partner/husband, summarized into No education (those who have never attended school and those in informal preschool), Basic education (those who have attended 0–4 grades or in school-based pre-primary class or have completed grade 8 at the lower basic level), and Secondary or Higher (completed grade 12 or above); 74 responses categorised as “Don’t know” have been classified into “No education”. |
| 17 | Time to reach the nearest health facility | A dichotomous variable, categorised into 30 minutes or less, and more than 30 minutes from their place of residence. |
| 18 | Used internet | Internet access status of the respondent, categorized into “Never used” and “Used”. |
| 19 | Own a mobile phone | Whether a respondent has her own mobile or not was categorised into “Yes” and “No”. |
| 20 | Wealth quintile | A composite index of household possessions, assets, and amenities, derived using principal component analysis, was grouped as Poorest, Poorer, Middle, Rich, and Richer. |
| 21 | Place of residence | Place of residence of respondents: Rural, Urban |
| 22 | Province | The provincial residence of respondents at the time of the survey: Koshi, Madhesh, Bagmati, Gandaki, Lumbini, Karnali, and Sudurpaschim. |
