# Supplementary material for: Supervised machine learning-based prediction of modern contraceptive use among sexually active women in Nepal
Source: PLOS Digit Health. 2026 Jul 13;5(7):e0001578. doi: 10.1371/journal.pdig.0001578 (PMC13362090; doi:10.1371/journal.pdig.0001578)
Supplement: S4 Table — This document presents the confusion matrix of the Random Forest model. (DOCX) [file pdig.0001578.s004.docx]

**Confusion Matrix of the Random Forest Model**

| **Actual** | **Predicted** | | |
| --- | --- | --- | --- |
|  | **No (Not Using)** | **Yes (Using)** | **Total** |
| **No** | 344 (TN) | 270 (FP) | 614 |
| **Yes** | 168 (FN) | 610 (TP) | 778 |
| **Total** | 512 | 880 | 1392 |
