# Supplementary material for: Supervised machine learning-based prediction of modern contraceptive use among sexually active women in Nepal
Source: PLOS Digit Health. 2026 Jul 13;5(7):e0001578. doi: 10.1371/journal.pdig.0001578 (PMC13362090; doi:10.1371/journal.pdig.0001578)
Supplement: Attachment — Submitted filename: Comments_ml_contra_plos_v01.docx [file pdig.0001578.s007.docx]

We would like to thank all the reviewers for their suggestions and recommendations for improving this manuscript. Based on the comments and suggestions, we have tried our best to address all the issues raised by the reviewers. Below are the Tables with the comments from the reviewers (in the first column) and our responses (in the second column) on improving the manuscript. Based on the comments, updates in the manuscript are made accordingly.

Thank you!

**Reviewer 1**

| **Reviewer Comment** | **Author Response** |
| --- | --- |
| **Title:** I think that the following words 'A study among sexually active female population in Nepal' is not needed. It can be merged with the first part of the title and country added. | The title has been revised to ***“Supervised Machine Learning-Based Prediction of Modern Contraceptive Use among Sexually Active Women in Nepal”***, improving clarity and conciseness as suggested. |
| **Abstract:** Line 16 – modern contraceptive use. | The wording in the abstract has been corrected to consistently use the term 'modern contraceptive use'. |
| **Author summary:** Line 35–37 needs to be reworded. It is the same as that in the abstract. | The author's summary has been rewritten while retaining the core message. |
| **Introduction:** Figure 1 would benefit from a more suitable axis to effectively highlight the differences. Indicators that are 0 can be removed. Statements on lines 68–70 and the figure do not align (e.g., increased uptake of injections and pills is not clearly visible). | Figure 1 has been redesigned with improved axes, removal of zero-valued indicators, and clearer visualization of trends (with two facets). The statements regarding female contraceptives have been updated. |
| Lines 93–95 appear unnecessary as they are generic and unrelated to the study's objective. | These statements have been removed as suggested. |
| **Methods:**   1. Would be good to state the independent variables. 2. Elaborate on how and what was summarized. Elaborate on the variables used to develop the composite variable. 3. The data processing section starts off generically. 4. How were these four models chosen? Were they based on the literature? | 1. The independent variables are included in the ‘Independent variables’ subsection. 2. Further details about variables categorization, summarization, and composite variables are updated in the supplementary document. 3. The data processing section has been rewritten. 4. We selected four supervised models based on their frequent use in reproductive health research. These models are a combination that allowed us to compare traditional statistical modeling with modern machine learning approaches. |
| **Results:**   1. Should start with the number of women included in the dataset. 2. All percentages should have ‘n’ stated. 3. How is basic education defined? 4. What are the definitions of media categories? 5. Tables 2 and 4 seem redundant. 6. Is Table 3 showing metrics for the test set? 7. Differences between accuracy and ROC are minimal—what does this indicate? 8. Variable names need not be in text. 9. Check figure numbers. 10. SHAP variables need to be self-explanatory. 11. What additional information does Figure 3 add? | 1. The information about the number of women and the prevalence of modern contraception among them has been added at the beginning of the ‘Results’ section. 2. Percentage and ‘n’ have been updated as suggested in the format: (n, xx%). 3. Details about education categories, including basic education is updated in the **Supplementary File**. 4. Regarding family planning advertisement categories, it has been summarized into “Have Access” and “No Access”. It has been updated in the **Supplementary File**. 5. Information in Table 2 and Table 4 has been updated in the corresponding sections, hence removed. 6. Previously (Table 3), Revised (Table 2) presents the evaluation metrics of each ML model. These metrics are obtained after evaluation of the fitted model on the test dataset. 7. The small differences between accuracy and ROC–AUC across all models indicate stable and consistent model performance across classification thresholds. Accuracy ranged from 66.10% to 68.60%, while ROC–AUC ranged from 71.20% to 73.60%, with a similar relative ranking of models under both metrics. This consistency suggests that the models’ discriminative ability is robust and not highly sensitive to the choice of probability cutoff. 8. Variable names are detailed below the figures for reference and removed from the write-up. 9. Figures are numbered sequentially. 10. Feature names in the plot are made self-explanatory. 11. Previously (Figure 3), Revised (Figure 5) presents a SHAP beeswarm plot, which provides case-level interpretability of the ML model. Unlike global variable importance measures that only indicate the overall ranking of predictors, the beeswarm plot illustrates the direction and magnitude of each predictor’s contribution to modern contraceptive use across individuals. Specifically, Figure 5 shows the top five predictors that increase and decrease the likelihood of modern contraceptive use, thereby revealing how high and low values of each variable influence the outcome. |
| **Discussion:**   1. Why were factors like distance from facility and media exposure not picked up by the model? 2. ML findings resemble conventional analysis—what is the added value and strength of this approach? | 1. Although distance to health facilities and media exposure were included in the models, they did not show high importance after training. Their effects are likely indirect and mediated through more proximal factors such as education, employment, fertility preferences, and women’s autonomy. In machine learning models, variables that do not add substantial predictive value beyond correlated features tend to receive lower importance. 2. The strength of the machine learning approach lies in its ability to capture non-linear relationships and interactions, rank predictors by their predictive contribution, and evaluate performance on independent test data without strict parametric assumptions. The use of SHAP further enhances interpretability by clearly quantifying each factor’s contribution to modern contraceptive use, thereby complementing conventional regression analyses. |
| **Conclusion:** Is a repetition of results. What does this study add, and what are future recommendations? | The Conclusion has been revised to emphasize the added value of the study, highlighting how machine learning and SHAP analysis identify and prioritize key predictors beyond conventional methods. It also outlines policy implications for targeted interventions and future research directions, including model validation and qualitative studies to understand contraceptive behaviour better. |

**Reviewer 2**

| **Reviewer Comment** | **Author Response** |
| --- | --- |
| There is no discussion of feature standardization or potential collinearity among predictors such as age, education, number of sons, and wealth. This complicates SHAP interpretation. | 1. We assessed multicollinearity among predictors using the Variance Inflation Factor (VIF) on the training dataset. The mean VIF across predictors was approximately 4, indicating low overall multicollinearity. Some categorical indicators exhibited higher VIF values, reflecting expected correlations among related dummy variables. It has been discussed in the manuscript. 2. Regarding feature standardization, tree-based ensemble models (Random Forest and XGBoost) are robust to multicollinearity and do not require scaling, so all variables were retained for these models. For Logistic Regression and SVM, standardization was applied to numeric predictors within the pipeline to ensure proper model fitting. |
| The authors do not address class imbalance. Although the imbalance is mild (≈54%), weighting or stratified metrics should be discussed. | Although the class imbalance in our data is mild (≈54%), we have addressed it now, during model training, by applying the Synthetic Minority Oversampling Technique (SMOTE) to the training set only. |
| The study relies only on internal validation. External validation using NDHS 2016 or another dataset would strengthen generalizability. | We acknowledge the lack of external validation as a limitation. |
| SHAP interpretation is descriptive and does not imply causality. Language suggesting determinants may be overstated. | The manuscript has been revised to avoid causal language, clearly stating that SHAP values indicate associations and predictive contribution, not causation. |
| 1. Bayesian hyperparameter tuning is not fully explained. Parameter ranges, priors, and convergence details are missing. 2. Performance differences are small, so claims of superiority should be made cautiously. | 1. We have revised the Hyperparameter Tuning section to clearly describe the Bayesian optimization process, including the hyperparameters tuned (n_estimators, max_depth, min_samples_split, min_samples_leaf, max_features), the search ranges, 20 iterations, 10-fold stratified cross-validation, and ROC–AUC as the optimization metric. The final optimized parameters are explicitly reported, and the model was refitted and evaluated on an independent test set to ensure transparency, reproducibility, and clarity regarding the tuning process. 2. Based on the updated results, the performance differences among the models are small: AUC ranged from 71.2% (LR) to 73.6% (RF) and accuracy from 66.1% (LR) to 68.6% (RF). Accordingly, we have tempered claims of superiority, noting that while Random Forest had slightly higher AUC and accuracy, all models performed comparably, and the choice of Random Forest was made for its balance of predictive performance and interpretability using SHAP. |

**Reviewer 3**

| **Reviewer Comment** | **Author Response** |
| --- | --- |
| Model performance is modest compared to recent studies using XGBoost or LightGBM in similar settings. | This likely reflects differences in data context, sample characteristics, and feature sets. Based on the balanced dataset, Random Forest achieved slightly higher AUC (73.6%) and accuracy (68.6%) compared to other models, with XGBoost performing similarly. |
| The study lacks external validation across time periods or regions. | We acknowledge the lack of external validation as a limitation. |
| The negative association between education and contraceptive use is not sufficiently explored.  Son preference and gender dynamics require deeper discussion. | We have expanded the discussion to highlight that previous studies in Nepal have also observed a lower uptake of modern contraceptives among more educated women. In the Conclusion, we note that this finding points to the need for qualitative and mixed-methods research to better understand the social, cultural, and psychological factors influencing contraceptive preferences in this group.  We have discussed son preference and gender dynamics, highlighting how women’s fertility intentions and the sex composition of existing children shape contraceptive use in Nepal. Our findings, consistent with previous studies, show that having at least one son increases the likelihood of modern contraceptive use, reflecting the persistent influence of gender norms and son preference on reproductive decision-making. |
| Justification for choosing RF over XGB, rationale for 80/20 split, discussion of class balance, and clearer SHAP explanations are needed. | We added notes on the model selection process, the 80/20 split ensured sufficient training and test data, class imbalance was addressed with SMOTE, and SHAP magnitudes now clearly indicate the relative contribution of each predictor. |
| Abstract should include quantitative SHAP values or effect magnitudes. | The abstract now includes representative SHAP values and clearer quantitative statements of model performance. |
| Improve flow between sections, especially between model interpretation and discussion. | Transitions between sections are revised to improve coherence and narrative flow. |
| Add supplementary materials:   1. Full SHAP values 2. Confusion matrices 3. Correlation matrix 4. ROC curves for all models. | The supplementary materials are provided in word document and csv files. |
