# Supplementary material for: Supervised machine learning-based prediction of modern contraceptive use among sexually active women in Nepal
Source: PLOS Digit Health. 2026 Jul 13;5(7):e0001578. doi: 10.1371/journal.pdig.0001578 (PMC13362090; doi:10.1371/journal.pdig.0001578)
Supplement: Attachment — Submitted filename: Comments_ml_contra_plos_v02.docx [file pdig.0001578.s008.docx]

We would like to thank the reviewers for their valuable comments and constructive suggestions, which have helped improve the quality and clarity of this manuscript. We have carefully addressed all the issues raised and revised the manuscript accordingly. Below, we provide a detailed, point-by-point response to Reviewer 3, with reviewer comments presented alongside our responses.

**Reviewer 3**

| **Reviewer Comment** | **Author Response** |
| --- | --- |
| No automated feature selection method was applied before or during model training. | The Boruta feature selection algorithm was applied prior to model training. From 44 encoded features derived from 21 variables, Boruta identified 23 important (confirmed and tentative) features corresponding to 19 variables. Model training and evaluation were conducted using this reduced feature set. This clarification has now been added to the Methods section. |
| A brief paragraph in the Discussion comparing the study's AUC with the cited studies and explaining why more advanced methods were not attempted (or why results may differ due to Nepal's sociocultural context) would strengthen the response. | A paragraph has been added to the Discussion comparing the model’s AUC with findings from similar studies conducted in African and South Asian settings using DHS data. The revised text also explains that the moderate AUC likely reflects the complexity of contraceptive behavior in Nepal, which is influenced by sociocultural factors—such as son preference, ethnic norms, and couple-level decision-making—that are not fully captured in survey data. |
| Causal language concern: “determinants” should be replaced with “predictors.” | We agree with the reviewer and have revised the manuscript accordingly, replacing “determinants” with “predictors” throughout to avoid causal interpretation. |
| Minor grammatical error in abstract: “having at least one children ever born” → “having at least one child ever born.” | Corrected as suggested. |
| Fix the typo | The identified typographical error has been corrected. |
