# Supplementary material for: Supervised machine learning-based prediction of modern contraceptive use among sexually active women in Nepal
Source: PLOS Digit Health. 2026 Jul 13;5(7):e0001578. doi: 10.1371/journal.pdig.0001578 (PMC13362090; doi:10.1371/journal.pdig.0001578)
Supplement: Peer review history

# Peer review: Decision Letter 0

**DOI:** 10.1371/journal.pdig.0001578.r001

**Authors:** Ziad El-Khatib, Wenjun Mao

25 Nov 2025

PDIG-D-25-00535

Using a supervised machine learning approach for the prediction of modern contraceptive use: A study among sexually active female population in Nepal

PLOS Digital Health

Dear Dr. Acharya,

Thank you for submitting your manuscript to PLOS Digital Health. After careful consideration, we feel that it has merit but does not fully meet PLOS Digital Health's publication criteria as it currently stands. Therefore, we invite you to submit a revised version of the manuscript that addresses the points raised during the review process.

Please submit your revised manuscript by Jan 24 2026 11:59PM. If you will need more time than this to complete your revisions, please reply to this message or contact the journal office at digitalhealth@plos.org. When you're ready to submit your revision, log on to https://www.editorialmanager.com/pdig/ and select the 'Submissions Needing Revision' folder to locate your manuscript file.

Please include the following items when submitting your revised manuscript:

* A rebuttal letter that responds to each point raised by the editor and reviewer(s). You should upload this letter as a separate file labeled 'Response to Reviewers'. This file does not need to include responses to any formatting updates and technical items listed in the 'Journal Requirements' section below.

* A marked-up copy of your manuscript that highlights changes made to the original version. You should upload this as a separate file labeled 'Revised Manuscript with Track Changes'.

* An unmarked version of your revised paper without tracked changes. You should upload this as a separate file labeled 'Manuscript'.

If you would like to make changes to your financial disclosure, competing interests statement, or data availability statement, please make these updates within the submission form at the time of resubmission. Guidelines for resubmitting your figure files are available below the reviewer comments at the end of this letter.

We look forward to receiving your revised manuscript.

Kind regards,

Wenjun Mao

Academic Editor

PLOS Digital Health

Wenjun Mao

Academic Editor

PLOS Digital Health

Leo Anthony Celi

Editor-in-Chief

PLOS Digital Health

orcid.org/0000-0001-6712-6626

**Journal Requirements:**

1. Please ensure that the Title in your manuscript and the Title in your online submission form are the same and written in standard English, if it is not written in English already.

2. Please provide separate figure files in .tif or .eps format.

For more information about figure files please see our guidelines:

https://journals.plos.org/digitalhealth/s/figures

https://journals.plos.org/digitalhealth/s/figures#loc-file-requirements

3. We have noticed that you have uploaded Supporting Information files, but you have not included a list of legends. Please add a full list of legends for your Supporting Information files after the references list.

4. If the reviewer comments include a recommendation to cite specific previously published works, please review and evaluate these publications to determine whether they are relevant and should be cited. There is no requirement to cite these works unless the editor has indicated otherwise.

**Additional Editor Comments:**

Thank you for submitting your manuscript .The reviewers and I agree that your work addresses an important and underexplored application of machine learning in family planning research within the Nepalese context. However, substantial revisions are necessary to enhance clarity, methodological rigor, and interpretive depth before the manuscript can be considered for publication.

[Note: HTML markup is below. Please do not edit.]

**Reviewers' Comments:**

Reviewer's Responses to Questions

**Comments to the Author**

1. Does this manuscript meet PLOS Digital Health’s publication criteria? Is the manuscript technically sound, and do the data support the conclusions? The manuscript must describe methodologically and ethically rigorous research with conclusions that are appropriately drawn based on the data presented.

Reviewer #1: Yes

Reviewer #2: Yes

Reviewer #3: Yes

**********

2. Has the statistical analysis been performed appropriately and rigorously?

Reviewer #1: Yes

Reviewer #2: Yes

Reviewer #3: Yes

**********

3. Have the authors made all data underlying the findings in their manuscript fully available (please refer to the Data Availability Statement at the start of the manuscript PDF file)?

The PLOS Data policy requires authors to make all data underlying the findings described in their manuscript fully available without restriction, with rare exception. The data should be provided as part of the manuscript or its supporting information, or deposited to a public repository. For example, in addition to summary statistics, the data points behind means, medians and variance measures should be available. If there are restrictions on publicly sharing data—e.g. participant privacy or use of data from a third party—those must be specified.

Reviewer #1: Yes

Reviewer #2: Yes

Reviewer #3: Yes

**********

4. Is the manuscript presented in an intelligible fashion and written in standard English?

PLOS Digital Health does not copyedit accepted manuscripts, so the language in submitted articles must be clear, correct, and unambiguous. Any typographical or grammatical errors should be corrected at revision, so please note any specific errors here.

Reviewer #1: Yes

Reviewer #2: Yes

Reviewer #3: Yes

**********

5. Review Comments to the Author

Please use the space provided to explain your answers to the questions above. You may also include additional comments for the author, including concerns about dual publication, research ethics, or publication ethics. (Please upload your review as an attachment if it exceeds 20,000 characters)

Reviewer #1: Thank you for your efforts on this manuscript.

Title: I think that the following words 'A study among sexually active female population in Nepal' is not needed. It can be merged with the first part of the title and country added.

Abstract: Line 16 - modern contraceptive use

Author summary: Line 35-37 needs to be reworded. It is the same as that in the abstract

Introduction: Figure 1 needs a better axis to highlight differences. The indicators that are 0 can be removed. The statement on lines 68-70 and the figure dont seem to align. For e.g. uptake of injections and pills has increased but this is not clearly visible in the figure.

Lines 93-95 dont seem to be needed. They are generic and not related to the study objective.

Methods: Would be good to state the independent variables. Data processing section starts off generically. This is not needed. Elaborate how and what was summarized. Elaborate on what variables were used to develop the composite variable. How were these 4 models chosen? Were they based on the literature?

Results: Should start with number of women included in the dataset. All % should also have n stated.How is basic education defined? What are the definitions of the various media categories? Table 2 and 4 seem redundant. Is Table 3 showing the metrics for the test set? The difference between accuracy and ROC among the models is minimal. What does this indicate? Variable names dont need to be part of the writeup. Please check figure numbers. For the SHAP figure, variables need to be made self explanatory. What additional information does figure 3 add?

Discussion: Why do you think factors like distance from facility and media exposure were not picked up by the model? The factors that are identified through ML are very similar what we know through conventional analysis. What is the added value of this approach? This is not coming out clearly. What were the strengths of this approach?

Conclusion: Is a repetition of results. What does this study add? What are future recommendations?

Reviewer #2: This manuscript presents some interesting findings and generally well excuted, however I have followung changes/ comments,

1) there’s no discussion of feature standardization or potential collinearity among predictors. Variables such as age, education, number of sons, and household wealth are likely to be correlated. Tree-based models can tolerate some multicollinearity, but it still complicates interpretation — especially when SHAP values are later used to describe feature importance.

2) the authors don’t address class imbalance. Modern contraceptive use accounts for about 54% of the sample, so the imbalance is mild, but it would still be good practice to mention whether any weighting or stratified metrics (e.g., precision, recall, or F1-score) were considered.

3) the paper relies entirely on internal validation through cross-validation within the same NDHS 2022 dataset. This is fine for initial modeling, but an external validation step — for example, using the 2016 DHS data or another regional dataset — would have provided a stronger test of generalizability.

The SHAP interpretation is also largely descriptive. The visualizations are informative, but SHAP doesn’t imply causality or control for confounding, so some of the language suggesting “determinants” may be overstated.

4) on the technical side, the Bayesian hyperparameter tuning is mentioned but not fully explained — the parameter ranges, priors, and convergence details are missing. Also, the performance differences among models are quite small (AUC variations under 2%), so the claim that one model “outperformed” the others should be presented more cautiously.

Reviewer #3: The study addresses a critical gap identified in recent literature: while digital health interventions for family planning have expanded rapidly, particularly through mobile platforms and telemedicine, the integration of advanced predictive modeling to identify at-risk populations remains underutilized in low- and middle-income countries like Nepal. This work contributes to the growing body of evidence demonstrating how machine learning can transform population-level survey data into actionable insights for targeted public health interventions. So, I congratulate the authors on an this important line of work and bringing focus to this important topic of research. However, there are a few issues that should be addressed:

1. The study relies on manual feature selection informed by literature. Recent work emphasizes the value of automated feature engineering and selection techniques, including recursive feature elimination or model-based importance ranking, which could strengthen the analysis.

2. While the RF model's 70.85% AUC is reasonable, recent comparable studies have achieved higher performance metrics. For example, a 2024 study (Melaku et al. 2025 in BMC Public Health or Yehuala, 2024 in BMC Health Serv Res) on East African family planning non-use achieved high accuracy and AUC using XGBoost and LightGBM with SHAP, and a 2025 study on Ethiopian fertility achieved similar high performance (Adem et al., BMJ Public Health). The relatively modest performance in this study may reflect the complexity of contraceptive behavior in Nepal's unique sociocultural context, but exploring more advanced techniques (ensemble stacking, deep learning, or addressing class imbalance with SMOTE) could potentially improve results.

3. The study lacks external validation on data from different time periods or regions within Nepal, limiting assessment of model generalizability.

4. Improve the discussion section to reflect on some of the important policy changes happening in Nepal - The finding that modern contraceptive use has stagnated at approximately 36% among sexually active women, despite national policy efforts, requires deeper analysis. Recent work by Dulal et al. (2024) (https://dhsprogram.com/pubs/pdf/FA155/FA155.pdf) examining DHS data from 2016-2022 identifies several concerning trends including increased use of traditional methods (nearly doubling) and declining reliance on male-focused methods. This shift suggests that

interventions may need to address not just access, but also quality of care, method satisfaction, and gender dynamics in contraceptive decision-making.

5. The paper mentions the unexpected negative relationship between education and contraceptive use but doesn't adequately explore this finding. A related work from Karki et al. (2024) from Reproductive Health found that while educational attainment has increased, reproductive health literacy remains limited, particularly regarding long-acting reversible contraceptives (LARCs). Educated women may thus have access to misinformation through digital media and social networks, leading to method avoidance? or similar findings in Shrestha et al., 2025 (10.1136/bmjph-2025-002576).

6. Kindly deepen the discussion on son preference and gender dynamics. Add discussion on possible confounders (spousal separation due to migration, cohort effects), preference for traditional methods among educated women, quality vs. quantity of reproductive health education. All of these are discussed in references: Dulal et al. 2024, Shrestha et al. 2025, Karki et al. 2024.

7. Why RF chosen was over XGB despite similar performance? Please provide some rationale for 80/20 split vs. other ratios. Write a short sentence discussion of class balance (53.68% vs. 46.32%). Similarly, the SHAP interpretations should be improved. Explanation of SHAP value magnitudes (what does 0.037 mean clinically?). Discussion of feature correlations affecting SHAP (education-wealth r=0.48?). Comparison: SHAP importance vs. traditional logistic regression coefficients.

8. Improve the abstract with specific SHAP values or effect magnitudes - "Age (SHAP=0.037) and number of sons (SHAP=0.036) were the strongest predictors" or "Model explains 71% of variation in contraceptive use decisions".

9. Improve transition and flow between a few sections: Between "Model interpretation" and "Discussion" (currently abrupt). IMprove flow between demographic findings and policy implications and also between limitations and future directions.

10. Add Supplementary Materials for the following:

a. Full SHAP values for all features (not just top 10)

b. Confusion matrix details

c. Feature correlation matrix

d. ROC curves for all 4 models (not just RF)

**********

6. PLOS authors have the option to publish the peer review history of their article (what does this mean?). If published, this will include your full peer review and any attached files.

**Do you want your identity to be public for this peer review?**  If you choose “no”, your identity will remain anonymous but your review may still be made public.

For information about this choice, including consent withdrawal, please see our Privacy Policy.

Reviewer #1: No

Reviewer #2: No

Reviewer #3: **Yes:** SAPTARSHI PURKAYASTHA

**********

[NOTE: If reviewer comments were submitted as an attachment file, they will be attached to this email and accessible via the submission site. Please log into your account, locate the manuscript record, and check for the action link "View Attachments". If this link does not appear, there are no attachment files.]

**Figure resubmission:**

While revising your submission, we strongly recommend that you use PLOS’s NAAS tool (https://ngplosjournals.pagemajik.ai/artanalysis) to test your figure files. NAAS can convert your figure files to the TIFF file type and meet basic requirements (such as print size, resolution), or provide you with a report on issues that do not meet our requirements and that NAAS cannot fix.

After uploading your figures to PLOS’s NAAS tool - https://ngplosjournals.pagemajik.ai/artanalysis, NAAS will process the files provided and display the results in the "Uploaded Files" section of the page as the processing is complete. If the uploaded figures meet our requirements (or NAAS is able to fix the files to meet our requirements), the figure will be marked as "fixed" above. If NAAS is unable to fix the files, a red "failed" label will appear above. When NAAS has confirmed that the figure files meet our requirements, please download the file via the download option, and include these NAAS processed figure files when submitting your revised manuscript.

**Reproducibility:**

To enhance the reproducibility of your results, we recommend that authors of applicable studies deposit laboratory protocols in protocols.io, where a protocol can be assigned its own identifier (DOI) such that it can be cited independently in the future. Additionally, PLOS ONE offers an option to publish peer-reviewed clinical study protocols. Read more information on sharing protocols at https://plos.org/protocols?utm_medium=editorial-email&utm_source=authorletters&utm_campaign=protocols

# Author comment: Author response to Decision Letter 1

**DOI:** 10.1371/journal.pdig.0001578.r002

12 Jan 2026

Attachment Submitted filename: Comments_ml_contra_plos_v01.docx

# Peer review: Decision Letter 1

**DOI:** 10.1371/journal.pdig.0001578.r003

**Authors:** Ziad El-Khatib, Wenjun Mao, Leo Anthony Celi, Wenjun Mao

19 Mar 2026

PDIG-D-25-00535R1

Supervised Machine Learning-Based Prediction of Modern Contraceptive Use among Sexually Active Women in Nepal

PLOS Digital Health

Dear Dr. Acharya,

Thank you for submitting your manuscript to PLOS Digital Health. After careful consideration, we feel that it has merit but does not fully meet PLOS Digital Health's publication criteria as it currently stands. Therefore, we invite you to submit a revised version of the manuscript that addresses the points raised during the review process.

Please submit your revised manuscript by Apr 18 2026 11:59PM. If you will need more time than this to complete your revisions, please reply to this message or contact the journal office at digitalhealth@plos.org. When you're ready to submit your revision, log on to https://www.editorialmanager.com/pdig/ and select the 'Submissions Needing Revision' folder to locate your manuscript file.

Please include the following items when submitting your revised manuscript:

* A letter that responds to each point raised by the editor and reviewer(s). You should upload this letter as a separate file labeled 'Response to Reviewers'. This file does not need to include responses to any formatting updates and technical items listed in the 'Journal Requirements' section below.

* A marked-up copy of your manuscript that highlights changes made to the original version. You should upload this as a separate file labeled 'Revised Manuscript with Track Changes'.

* An unmarked version of your revised paper without tracked changes. You should upload this as a separate file labeled 'Manuscript'.

If you would like to make changes to your financial disclosure, competing interests statement, or data availability statement, please make these updates within the submission form at the time of resubmission. Guidelines for resubmitting your figure files are available below the reviewer comments at the end of this letter.

We look forward to receiving your revised manuscript.

Kind regards,

Wenjun Mao

Academic Editor

PLOS Digital Health

Leo Anthony Celi

Editor-in-Chief

PLOS Digital Health

orcid.org/0000-0001-6712-6626

**Journal Requirements:**

If the reviewer comments include a recommendation to cite specific previously published works, please review and evaluate these publications to determine whether they are relevant and should be cited. There is no requirement to cite these works unless the editor has indicated otherwise.

**Additional Editor Comments (if provided):**

Thank you for submitting your manuscript to Plos digital health.After careful evaluation, the editorial team has concluded that the paper cannot be accepted in its present form, however, we invite you to submit a thoroughly revised version that fully addresses the comments from the reviewers.Please prepare a point-by-point rebuttal letter indicating precisely where and how each comment has been accommodated,track-changes or highlighted text must be included in the revised manuscript.

[Note: HTML markup is below. Please do not edit.]

**Reviewers' Comments:**

Reviewer's Responses to Questions

**Comments to the Author**

1. If the authors have adequately addressed your comments raised in a previous round of review and you feel that this manuscript is now acceptable for publication, you may indicate that here to bypass the “Comments to the Author” section, enter your conflict of interest statement in the “Confidential to Editor” section, and submit your "Accept" recommendation.

Reviewer #1: All comments have been addressed

Reviewer #3: (No Response)

**********

2. Does this manuscript meet PLOS Digital Health’s publication criteria? Is the manuscript technically sound, and do the data support the conclusions? The manuscript must describe methodologically and ethically rigorous research with conclusions that are appropriately drawn based on the data presented.

Reviewer #1: Yes

Reviewer #3: Yes

**********

3. Has the statistical analysis been performed appropriately and rigorously?

Reviewer #1: Yes

Reviewer #3: No

**********

4. Have the authors made all data underlying the findings in their manuscript fully available (please refer to the Data Availability Statement at the start of the manuscript PDF file)?

The PLOS Data policy requires authors to make all data underlying the findings described in their manuscript fully available without restriction, with rare exception. The data should be provided as part of the manuscript or its supporting information, or deposited to a public repository. For example, in addition to summary statistics, the data points behind means, medians and variance measures should be available. If there are restrictions on publicly sharing data—e.g. participant privacy or use of data from a third party—those must be specified.

Reviewer #1: Yes

Reviewer #3: Yes

**********

5. Is the manuscript presented in an intelligible fashion and written in standard English?

PLOS Digital Health does not copyedit accepted manuscripts, so the language in submitted articles must be clear, correct, and unambiguous. Any typographical or grammatical errors should be corrected at revision, so please note any specific errors here.

Reviewer #1: Yes

Reviewer #3: (No Response)

**********

6. Review Comments to the Author

Please use the space provided to explain your answers to the questions above. You may also include additional comments for the author, including concerns about dual publication, research ethics, or publication ethics. (Please upload your review as an attachment if it exceeds 20,000 characters)

**Reviewer #1:** (No Response)

**Reviewer #3:**  Thank you for addressing most comments and adding the supplementary material. The manuscript has improved readability with the improved flow and additional material to improve reproducibility. However, the main concern about feature selection hasn't been addressed, and a few other minor concerns:

The manuscript still has that "A total of 21 independent variables were manually selected from the dataset, informed by relevant literature and prior findings" (line 122). SHAP global feature importance is used post hoc to rank predictors (lines 291–303), but no automated feature selection method (recursive feature elimination, Boruta, LASSO-based selection, etc.) was applied before or during model training. There is no comparison of automated ML feature selection vs this manual feature selection, given that the justification for the literature and prior findings is weak for a social challenge.

Add a paragraph in the Methods justifying the manual feature selection approach and briefly explaining why automated methods (RFE, Boruta) were not used, or ideally run one automated method as a sensitivity check.

SMOTE and Bayesian tuning improve the methodology, but more advanced techniques is not explored or explicitly rebutted. A brief paragraph in the Discussion comparing the study's AUC with the cited studies and explaining why more advanced methods were not attempted (or why results may differ due to Nepal's sociocultural context) would strengthen the response.

Causal language concern: I had noted from Round 1, flagging overstated causal language. While the revision improves this, the word "determinants" still appears in the study workflow diagram (Fig 2: "Identify determinants of modern contraceptive use"). I suggest that the authors may want to change this to "predictors" for consistency with the rest of the manuscript.

Minor grammatical error in abstract: "having at least one children even born" -> "having at least one child ever born".

Fix the typo: "being employed (mean |SHAP| = 0.22)" appears in both the abstract and body. However, based on Figure 4, it shows 0.022 (or possibly 0.012, matching line 31 in the tracked-changes version which says "currently working (mean |SHAP| = 0.012)"). This is a 10x error that must be corrected.

**********

7. PLOS authors have the option to publish the peer review history of their article (what does this mean?). If published, this will include your full peer review and any attached files.

**Do you want your identity to be public for this peer review?**  If you choose “no”, your identity will remain anonymous but your review may still be made public.

For information about this choice, including consent withdrawal, please see our Privacy Policy.

Reviewer #1: No

Reviewer #3: **Yes:** Saptarshi Purkayastha

**********

[NOTE: If reviewer comments were submitted as an attachment file, they will be attached to this email and accessible via the submission site. Please log into your account, locate the manuscript record, and check for the action link "View Attachments". If this link does not appear, there are no attachment files.]

**Figure resubmission:**

While revising your submission, we strongly recommend that you use PLOS’s NAAS tool (https://ngplosjournals.pagemajik.ai/artanalysis) to test your figure files. NAAS can convert your figure files to the TIFF file type and meet basic requirements (such as print size, resolution), or provide you with a report on issues that do not meet our requirements and that NAAS cannot fix.

After uploading your figures to PLOS’s NAAS tool - https://ngplosjournals.pagemajik.ai/artanalysis, NAAS will process the files provided and display the results in the "Uploaded Files" section of the page as the processing is complete. If the uploaded figures meet our requirements (or NAAS is able to fix the files to meet our requirements), the figure will be marked as "fixed" above. If NAAS is unable to fix the files, a red "failed" label will appear above. When NAAS has confirmed that the figure files meet our requirements, please download the file via the download option, and include these NAAS processed figure files when submitting your revised manuscript.

**Reproducibility:**

To enhance the reproducibility of your results, we recommend that authors of applicable studies deposit laboratory protocols in protocols.io, where a protocol can be assigned its own identifier (DOI) such that it can be cited independently in the future. Additionally, PLOS ONE offers an option to publish peer-reviewed clinical study protocols. Read more information on sharing protocols at https://plos.org/protocols?utm_medium=editorial-email&utm_source=authorletters&utm_campaign=protocols

# Author comment: Author response to Decision Letter 2

**DOI:** 10.1371/journal.pdig.0001578.r004

23 Apr 2026

Attachment Submitted filename: Comments_ml_contra_plos_v02.docx

# Peer review: Decision Letter 2

**DOI:** 10.1371/journal.pdig.0001578.r005

**Authors:** Ziad El-Khatib, Wenjun Mao, Leo Anthony Celi, Wenjun Mao, Leo Anthony Celi, Wenjun Mao

26 Jun 2026

Supervised Machine Learning-Based Prediction of Modern Contraceptive Use among Sexually Active Women in Nepal

PDIG-D-25-00535R2

Dear Mr Acharya,

We are pleased to inform you that your manuscript 'Supervised Machine Learning-Based Prediction of Modern Contraceptive Use among Sexually Active Women in Nepal' has been provisionally accepted for publication in PLOS Digital Health.

Before your manuscript can be formally accepted you will need to complete some formatting changes, which you will receive in a follow-up email from a member of our team.

Please note that your manuscript will not be scheduled for publication until you have made the required changes, so a swift response is appreciated.

IMPORTANT: The editorial review process is now complete. PLOS will only permit corrections to spelling, formatting or significant scientific errors from this point onwards. Requests for major changes, or any which affect the scientific understanding of your work, will cause delays to the publication date of your manuscript.

If your institution or institutions have a press office, please notify them about your upcoming paper to help maximize its impact. If they'll be preparing press materials, please inform our press team as soon as possible -- no later than 48 hours after receiving the formal acceptance. Your manuscript will remain under strict press embargo until 2 pm Eastern Time on the date of publication. For more information, please contact digitalhealth@plos.org.

Thank you again for supporting Open Access publishing; we are looking forward to publishing your work in PLOS Digital Health.

Best regards,

Wenjun Mao

Academic Editor

PLOS Digital Health

***********************************************************

**Additional Editor Comments (if provided):**

We are pleased to inform you that your manuscript has been accepted for publication. The reviewers found it to be a high-quality and valuable contribution to the field. We will be in touch shortly regarding the next steps in the publication process.

**Reviewer Comments (if any, and for reference):**

Reviewer's Responses to Questions

**Comments to the Author**

1. If the authors have adequately addressed your comments raised in a previous round of review and you feel that this manuscript is now acceptable for publication, you may indicate that here to bypass the “Comments to the Author” section, enter your conflict of interest statement in the “Confidential to Editor” section, and submit your "Accept" recommendation.

Reviewer #1: All comments have been addressed

Reviewer #3: All comments have been addressed

**********

2. Does this manuscript meet PLOS Digital Health’s publication criteria? Is the manuscript technically sound, and do the data support the conclusions? The manuscript must describe methodologically and ethically rigorous research with conclusions that are appropriately drawn based on the data presented.

Reviewer #1: Yes

Reviewer #3: Yes

**********

3. Has the statistical analysis been performed appropriately and rigorously?

Reviewer #1: Yes

Reviewer #3: Yes

**********

4. Have the authors made all data underlying the findings in their manuscript fully available (please refer to the Data Availability Statement at the start of the manuscript PDF file)?

The PLOS Data policy requires authors to make all data underlying the findings described in their manuscript fully available without restriction, with rare exception. The data should be provided as part of the manuscript or its supporting information, or deposited to a public repository. For example, in addition to summary statistics, the data points behind means, medians and variance measures should be available. If there are restrictions on publicly sharing data—e.g. participant privacy or use of data from a third party—those must be specified.

Reviewer #1: Yes

Reviewer #3: Yes

**********

5. Is the manuscript presented in an intelligible fashion and written in standard English?

PLOS Digital Health does not copyedit accepted manuscripts, so the language in submitted articles must be clear, correct, and unambiguous. Any typographical or grammatical errors should be corrected at revision, so please note any specific errors here.

Reviewer #1: Yes

Reviewer #3: Yes

**********

6. Review Comments to the Author

Please use the space provided to explain your answers to the questions above. You may also include additional comments for the author, including concerns about dual publication, research ethics, or publication ethics. (Please upload your review as an attachment if it exceeds 20,000 characters)

Reviewer #1: (No Response)

Reviewer #3: The authors address all the previous comments in this version.

**********

7. PLOS authors have the option to publish the peer review history of their article (what does this mean?). If published, this will include your full peer review and any attached files.

**Do you want your identity to be public for this peer review?**  If you choose “no”, your identity will remain anonymous but your review may still be made public.

For information about this choice, including consent withdrawal, please see our Privacy Policy.

Reviewer #1: No

Reviewer #3: **Yes:** Saptarshi Purkayastha

**********
